# Bis[1-(eth­oxy­carbonyl­meth­yl)pyridinium] bis­(1,2-dicyano­ethene-1,2-dithiol­ato-κ^2^ *S*,*S*′)nickelate(II)

**DOI:** 10.1107/S1600536812033831

**Published:** 2012-08-04

**Authors:** Tian-li Ren, Cheng-jian Yang, De-yu Fang, Guo-xun Shi, Xue Zhu

**Affiliations:** a Department of Immunology and Rheumatology, the Second People’s Hospital of Wuxi, Wuxi 214002, People’s Republic of China; b Key Laboratory of Nuclear Medicine, Ministry of Health, Jiangsu Key Laboratory of Molecular Nuclear Medicine, Jiangsu Institute of Nuclear Medicine. Wuxi 214063, People’s Republic of China

## Abstract

The asymmetric unit of the title ion-pair complex, (C_9_H_12_NO_2_)_2_[Ni(C_4_N_2_S_2_)_2_], contains two 1-(eth­oxy­carbonyl­meth­yl)pyridinium cations and one bis­(1,2-dicyano­ethene-1,2-dithiol­ato)nickelate(II) dianion, which exhibits a slightly distorted square-planar coordination geometry. In the crystal, the cations are linked by strong C—H⋯O hydrogen bonds into *C*(6) chains along [100]. The cations and anions are linked into a three-dimensional architecture by weak C—H⋯N and C—H⋯S inter­actions.

## Related literature

For details of other maleonitrile­dithiol­ate metal complexes, see: Robertson & Cronin (2002 ▶); Coomber *et al.* (1996 ▶); Duan *et al.* (2010 ▶); Wang *et al.* (2012 ▶). For general background to the use of maleonitrile­dithiol­ate transition metal complexes as building blocks for optical, magnetic and conducting mol­ecular materials, see: Brammer (2004 ▶); Ni *et al.* (2005 ▶); Robin & Fromm (2006 ▶). For graph-set notation, see: Bernstein *et al.* (1995 ▶).
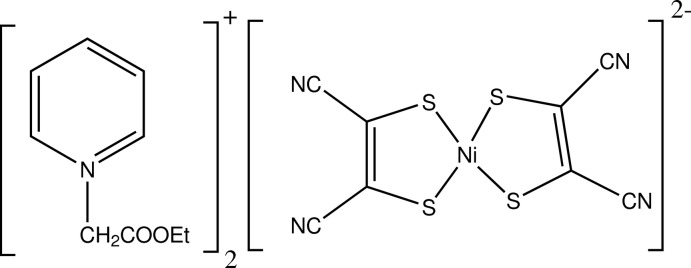


## Experimental

### 

#### Crystal data

(C_9_H_12_NO_2_)_2_[Ni(C_4_N_2_S_2_)_2_]
*M* *_r_* = 671.46
Monoclinic, 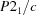

*a* = 9.486 (2) Å
*b* = 19.542 (5) Å
*c* = 17.635 (4) Å
β = 104.803 (4)°
*V* = 3160.6 (13) Å^3^
*Z* = 4
Mo *K*α radiation
μ = 0.92 mm^−1^
*T* = 291 K
0.30 × 0.15 × 0.10 mm

#### Data collection

Bruker SMART APEX CCD diffractometer
Absorption correction: multi-scan (*SADABS*; Bruker, 2000 ▶) *T*_min_ = 0.770, *T*_max_ = 0.914
16888 measured reflections
6200 independent reflections
5024 reflections with *I* > 2σ(*I*)
*R*_int_ = 0.026

#### Refinement

*R*[*F* ^2^ > 2σ(*F* ^2^)] = 0.043
*wR*(*F* ^2^) = 0.114
*S* = 1.02
6200 reflections
370 parameters
H-atom parameters constrained
Δρ_max_ = 0.18 e Å^−3^
Δρ_min_ = −0.34 e Å^−3^

### 

Data collection: *SMART* (Bruker, 2000 ▶); cell refinement: *SAINT* (Bruker, 2000 ▶); data reduction: *SAINT*; program(s) used to solve structure: *SHELXTL* (Sheldrick, 2008 ▶); program(s) used to refine structure: *SHELXTL*; molecular graphics: *SHELXTL*; software used to prepare material for publication: *SHELXTL*.

## Supplementary Material

Crystal structure: contains datablock(s) I, global. DOI: 10.1107/S1600536812033831/bx2420sup1.cif

Structure factors: contains datablock(s) I. DOI: 10.1107/S1600536812033831/bx2420Isup2.hkl

Additional supplementary materials:  crystallographic information; 3D view; checkCIF report

## Figures and Tables

**Table 1:** Hydrogen-bond geometry (Å, °)

*D*—H⋯*A*	*D*—H	H⋯*A*	*D*⋯*A*	*D*—H⋯*A*
C10—H10*A*⋯O1^i^	0.96	2.13	3.064 (4)	164
C13—H13*A*⋯N6^ii^	0.96	2.57	3.357 (4)	140
C15—H15*B*⋯S2	0.96	2.86	3.680 (3)	145

Symmetry codes: (i) 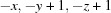; (ii) 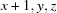.

**Fig. 1.**
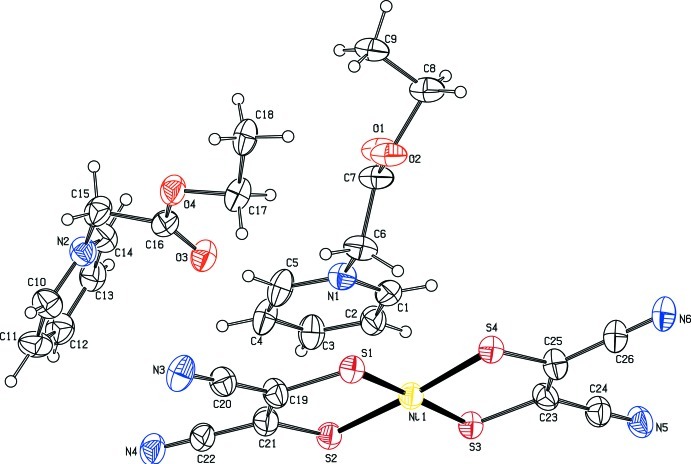
The asymmetric unit of the title compound with atom labels. Displacement ellipsoids were draw at the 30% probability.

**Fig. 2.**
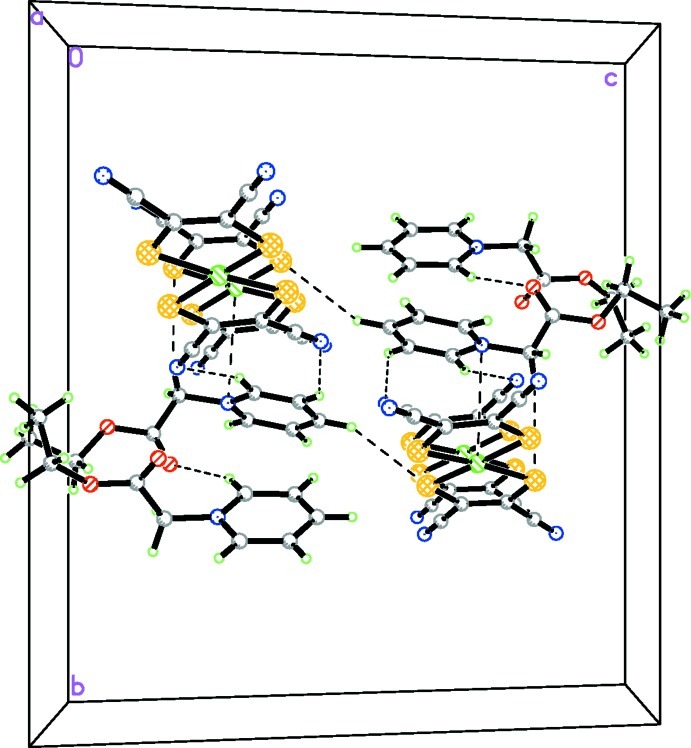
The packing diagram of title complex as viewed along a axis. (dotted lines are H-bonds between cations and anions)

## supplementary crystallographic information

### Comment

The maleonitriledithiolate (or 1,2-dicyanoethene-1,2-dithiolate, mnt^2-^) transition metal complexes, which is one typical kind of bis-1,2-dithiolene complexes, are often used as building blocks for optical, magnetic and conducting molecular materials (Brammer, 2004; Robin & Fromm, 2006; Duan *et al.*, 2010). It has been found that maleonitriledithiolate (mnt^2-^) complexes are charge-transfer salts, and they have various structure and the intra- or inter- molecular contacts which can result in large changes of physical properties of the complexes based on the [*M*(mnt)_2_]^n-^ (Robertson & Cronin, 2002; Coomber *et al.*, 1996; Ni *et al.*, 2005; Duan *et al.*, 2010; Wang *et al.*, 2012). We report here a new [Ni(mnt)_2_]^2-^ salt containing the 1-(ethoxycarbonylmethyl)pyridinium cation, (EtOAcPy)^+^, as shown in Fig. 1. The asymmetric unit contains two (EtOAcPy)^+^cation and one [Ni(mnt)_2_^2-^] dianion. The [Ni(mnt)_2_]^2-^ dianion exhibits a slightly distorted square-planar coordination geometry. In the crystal, cations are linked by strong C—H···O hydrogen bond into chains with graph-set notation *C*(6) along [100] (Bernstein *et al.*, 1995). The cations and anions are linked by weak C—H ··· N and C—H··· S interactions (Table 1).

### Experimental

The title compound was prepared by the direct reaction of NiCl_2_.6H_2_O, Na_2_mnt and (EtOAcPy)^+^.Br^-^ in the mixed solution of ethanol and H_2_O (1:1). Red–brown block-like single crystals were obtained by slow evaporation of the acetonitrile solution at room temperature for about one week.

### Refinement

All H atoms were fixed geometrically and treated as riding with C—H = 0.96 Å (methyl), 0.96 Å (methylene) and 0.96 Å (pyridyl), with *U*iso(H) = 1.2*U*eq (pyridyl) or *U*iso(H) = 1.5*U*eq (methyl and methylene).

### Crystal data

**Table:**

(C_9_H_12_NO_2_)_2_[Ni(C_4_N_2_S_2_)_2_]	*F*(000) = 1384
*M**_r_* = 671.46	*D*_x_ = 1.411 Mg m^−^^3^
Monoclinic, *P*2_1_/*c*	Mo *K*α radiation, λ = 0.71073 Å
Hall symbol: -P 2ybc	Cell parameters from 1842 reflections
*a* = 9.486 (2) Å	θ = 2.4–20.0°
*b* = 19.542 (5) Å	µ = 0.92 mm^−^^1^
*c* = 17.635 (4) Å	*T* = 291 K
β = 104.803 (4)°	Block, red-brown
*V* = 3160.6 (13) Å^3^	0.30 × 0.15 × 0.10 mm
*Z* = 4	

### Data collection

**Table:**

Bruker SMART APEX CCD diffractometer	6200 independent reflections
Radiation source: sealed tube	5024 reflections with *I* > 2σ(*I*)
Graphite monochromator	*R*_int_ = 0.026
phi and ω scans	θ_max_ = 26.0°, θ_min_ = 2.1°
Absorption correction: multi-scan (*SADABS*; Bruker, 2000)	*h* = −11→11
*T*_min_ = 0.770, *T*_max_ = 0.914	*k* = −24→24
16888 measured reflections	*l* = −21→12

### Refinement

**Table:**

Refinement on *F*^2^	Primary atom site location: structure-invariant direct methods
Least-squares matrix: full	Secondary atom site location: difference Fourier map
*R*[*F*^2^ > 2σ(*F*^2^)] = 0.043	Hydrogen site location: inferred from neighbouring sites
*wR*(*F*^2^) = 0.114	H-atom parameters constrained
*S* = 1.02	*w* = 1/[σ^2^(*F*_o_^2^) + (0.06*P*)^2^ + 1.22*P*] where *P* = (*F*_o_^2^ + 2*F*_c_^2^)/3
6200 reflections	(Δ/σ)_max_ < 0.001
370 parameters	Δρ_max_ = 0.18 e Å^−^^3^
0 restraints	Δρ_min_ = −0.34 e Å^−^^3^

### Special details

**Table:**

Geometry. All e.s.d.'s (except the e.s.d. in the dihedral angle between two l.s. planes) are estimated using the full covariance matrix. The cell e.s.d.'s are taken into account individually in the estimation of e.s.d.'s in distances, angles and torsion angles; correlations between e.s.d.'s in cell parameters are only used when they are defined by crystal symmetry. An approximate (isotropic) treatment of cell e.s.d.'s is used for estimating e.s.d.'s involving l.s. planes.
Refinement. Refinement of *F*^2^ against ALL reflections. The weighted *R*-factor *wR* and goodness of fit *S* are based on *F*^2^, conventional *R*-factors *R* are based on *F*, with *F* set to zero for negative *F*^2^. The threshold expression of *F*^2^ > σ(*F*^2^) is used only for calculating *R*-factors(gt) *etc*. and is not relevant to the choice of reflections for refinement. *R*-factors based on *F*^2^ are statistically about twice as large as those based on *F*, and *R*- factors based on ALL data will be even larger.

### Fractional atomic coordinates and isotropic or equivalent isotropic displacement parameters (Å^2^)

**Table:**

	*x*	*y*	*z*	*U*_iso_*/*U*_eq_	
C1	0.0092 (3)	0.27127 (15)	0.68082 (18)	0.0574 (7)	
H1A	−0.0512	0.2503	0.7105	0.069*	
C2	−0.0336 (3)	0.27156 (17)	0.60181 (19)	0.0627 (8)	
H2A	−0.1235	0.2499	0.5752	0.075*	
C3	0.0489 (3)	0.30165 (17)	0.5601 (2)	0.0659 (8)	
H3A	0.0172	0.3019	0.5038	0.079*	
C4	0.1749 (4)	0.33078 (17)	0.5965 (2)	0.0702 (9)	
H4A	0.2352	0.3526	0.5674	0.084*	
C5	0.2169 (4)	0.33164 (17)	0.67691 (19)	0.0690 (9)	
H5A	0.3086	0.3513	0.7042	0.083*	
C6	0.1778 (4)	0.29897 (17)	0.80314 (17)	0.0625 (8)	
H6A	0.2808	0.3076	0.8195	0.075*	
H6B	0.1605	0.2538	0.8202	0.075*	
C7	0.1028 (4)	0.35080 (17)	0.8425 (2)	0.0656 (8)	
C8	0.0589 (4)	0.37959 (17)	0.9706 (2)	0.0664 (9)	
H8A	0.0711	0.3532	1.0178	0.080*	
H8B	−0.0422	0.3875	0.9456	0.080*	
C9	0.1514 (4)	0.44077 (17)	0.99563 (19)	0.0657 (8)	
H9A	0.1085	0.4676	1.0295	0.099*	
H9B	0.2469	0.4261	1.0239	0.099*	
H9C	0.1585	0.4679	0.9514	0.099*	
C10	0.2726 (4)	0.56883 (17)	0.31523 (19)	0.0632 (8)	
H10A	0.1770	0.5767	0.2817	0.076*	
C11	0.3033 (4)	0.58304 (16)	0.3941 (2)	0.0678 (9)	
H11A	0.2314	0.6030	0.4171	0.081*	
C12	0.4402 (4)	0.56894 (17)	0.4396 (2)	0.0664 (8)	
H12A	0.4628	0.5784	0.4948	0.080*	
C13	0.5375 (3)	0.54072 (16)	0.40850 (19)	0.0608 (7)	
H13A	0.6330	0.5314	0.4413	0.073*	
C14	0.5035 (3)	0.52757 (16)	0.33203 (18)	0.0605 (7)	
H14A	0.5737	0.5057	0.3094	0.073*	
C15	0.3445 (4)	0.53147 (17)	0.20264 (18)	0.0658 (8)	
H15A	0.2409	0.5290	0.1813	0.079*	
H15B	0.3862	0.4883	0.1940	0.079*	
C16	0.4074 (3)	0.58706 (15)	0.16235 (18)	0.0580 (7)	
C17	0.4174 (4)	0.62653 (17)	0.0371 (2)	0.0668 (9)	
H17A	0.5182	0.6357	0.0617	0.080*	
H17B	0.3640	0.6688	0.0297	0.080*	
C18	0.4040 (4)	0.59306 (18)	−0.03955 (19)	0.0701 (9)	
H18A	0.4414	0.6207	−0.0750	0.105*	
H18B	0.4569	0.5507	−0.0301	0.105*	
H18C	0.3025	0.5838	−0.0622	0.105*	
C19	0.5736 (3)	0.30442 (17)	0.31581 (18)	0.0586 (7)	
C20	0.7122 (3)	0.27502 (17)	0.34896 (18)	0.0584 (7)	
C21	0.5209 (3)	0.30770 (16)	0.23727 (18)	0.0600 (7)	
C22	0.6003 (3)	0.27884 (16)	0.18676 (19)	0.0604 (7)	
C23	−0.0312 (3)	0.43365 (16)	0.27582 (17)	0.0548 (7)	
C24	−0.1682 (3)	0.46578 (17)	0.24053 (18)	0.0613 (7)	
C25	0.0161 (3)	0.42632 (17)	0.35483 (17)	0.0570 (7)	
C26	−0.0736 (3)	0.44181 (17)	0.40549 (19)	0.0614 (8)	
N1	0.1339 (3)	0.30149 (13)	0.71817 (14)	0.0553 (6)	
N2	0.3757 (3)	0.54281 (13)	0.28536 (14)	0.0578 (6)	
N3	0.8255 (3)	0.25235 (14)	0.37599 (17)	0.0700 (8)	
N4	0.6599 (3)	0.25557 (15)	0.14504 (16)	0.0668 (7)	
N5	−0.2783 (3)	0.49033 (15)	0.21326 (16)	0.0651 (7)	
N6	−0.1470 (3)	0.45396 (14)	0.44550 (16)	0.0645 (7)	
Ni1	0.27080 (4)	0.370117 (18)	0.29545 (2)	0.05072 (12)	
O1	0.0043 (3)	0.38524 (11)	0.80562 (13)	0.0695 (6)	
O2	0.1514 (2)	0.34855 (11)	0.91666 (13)	0.0687 (6)	
O3	0.4846 (2)	0.63080 (11)	0.19261 (12)	0.0618 (5)	
O4	0.3596 (2)	0.57755 (11)	0.08488 (12)	0.0634 (5)	
S1	0.46957 (8)	0.33440 (4)	0.37583 (4)	0.05255 (18)	
S2	0.35111 (8)	0.34322 (4)	0.19505 (4)	0.05189 (17)	
S3	0.07449 (8)	0.40886 (4)	0.21465 (4)	0.05150 (18)	
S4	0.18471 (8)	0.38983 (4)	0.39552 (4)	0.05204 (17)	

### Atomic displacement parameters (Å^2^)

**Table:**

	*U*^11^	*U*^22^	*U*^33^	*U*^12^	*U*^13^	*U*^23^
C1	0.0555 (17)	0.0526 (16)	0.0653 (18)	−0.0082 (13)	0.0176 (14)	−0.0130 (14)
C2	0.0542 (17)	0.0706 (19)	0.0645 (19)	−0.0201 (15)	0.0173 (14)	−0.0173 (15)
C3	0.0540 (17)	0.076 (2)	0.0641 (19)	−0.0093 (15)	0.0080 (14)	0.0125 (16)
C4	0.076 (2)	0.0659 (19)	0.0649 (19)	−0.0291 (17)	0.0112 (16)	0.0117 (16)
C5	0.069 (2)	0.0648 (19)	0.0645 (19)	−0.0179 (16)	0.0003 (15)	0.0222 (16)
C6	0.0695 (19)	0.0634 (18)	0.0540 (17)	0.0206 (15)	0.0143 (14)	−0.0037 (14)
C7	0.0636 (19)	0.0605 (18)	0.069 (2)	0.0149 (15)	0.0098 (16)	−0.0188 (16)
C8	0.0626 (19)	0.0677 (19)	0.0623 (18)	0.0139 (15)	0.0036 (15)	−0.0193 (15)
C9	0.069 (2)	0.0657 (19)	0.0630 (18)	0.0146 (15)	0.0184 (15)	−0.0181 (15)
C10	0.0568 (18)	0.0680 (19)	0.0641 (19)	0.0133 (15)	0.0142 (14)	0.0103 (15)
C11	0.076 (2)	0.0582 (18)	0.070 (2)	0.0250 (16)	0.0204 (17)	0.0048 (15)
C12	0.067 (2)	0.070 (2)	0.0600 (18)	−0.0121 (16)	0.0110 (15)	−0.0047 (15)
C13	0.0517 (16)	0.0602 (17)	0.068 (2)	−0.0049 (14)	0.0108 (14)	0.0073 (15)
C14	0.0603 (18)	0.0642 (18)	0.0600 (18)	0.0098 (14)	0.0207 (14)	0.0131 (14)
C15	0.077 (2)	0.0613 (18)	0.0574 (17)	−0.0215 (16)	0.0140 (15)	−0.0099 (14)
C16	0.0610 (18)	0.0534 (16)	0.0593 (17)	−0.0091 (14)	0.0150 (14)	−0.0094 (14)
C17	0.068 (2)	0.0584 (18)	0.069 (2)	−0.0180 (15)	0.0078 (16)	0.0077 (15)
C18	0.075 (2)	0.069 (2)	0.0642 (19)	−0.0213 (16)	0.0149 (16)	0.0186 (16)
C19	0.0442 (15)	0.0707 (19)	0.0624 (18)	0.0064 (13)	0.0161 (13)	0.0019 (15)
C20	0.0495 (17)	0.0670 (18)	0.0581 (17)	0.0027 (14)	0.0124 (13)	−0.0007 (14)
C21	0.0587 (17)	0.0632 (18)	0.0604 (18)	0.0127 (14)	0.0197 (14)	0.0052 (14)
C22	0.0602 (18)	0.0597 (17)	0.0644 (18)	0.0173 (14)	0.0216 (15)	0.0124 (14)
C23	0.0404 (14)	0.0676 (18)	0.0581 (17)	0.0016 (12)	0.0156 (12)	0.0073 (14)
C24	0.0545 (18)	0.0698 (19)	0.0603 (18)	0.0118 (15)	0.0162 (14)	0.0024 (15)
C25	0.0513 (16)	0.0680 (18)	0.0525 (16)	0.0062 (13)	0.0147 (13)	−0.0072 (14)
C26	0.0574 (17)	0.0676 (19)	0.0597 (18)	0.0142 (15)	0.0161 (14)	−0.0068 (15)
N1	0.0528 (13)	0.0582 (14)	0.0528 (14)	0.0015 (11)	0.0095 (11)	−0.0033 (11)
N2	0.0492 (14)	0.0653 (15)	0.0577 (14)	−0.0080 (11)	0.0111 (11)	−0.0050 (12)
N3	0.0570 (15)	0.0670 (16)	0.0742 (18)	0.0118 (13)	−0.0050 (13)	−0.0157 (14)
N4	0.0655 (16)	0.0733 (17)	0.0619 (16)	0.0269 (14)	0.0173 (13)	0.0104 (13)
N5	0.0482 (14)	0.0825 (18)	0.0672 (16)	0.0142 (13)	0.0193 (12)	0.0097 (14)
N6	0.0625 (16)	0.0694 (16)	0.0616 (15)	0.0164 (13)	0.0158 (13)	−0.0141 (13)
Ni1	0.0489 (2)	0.0502 (2)	0.0536 (2)	0.00300 (15)	0.01412 (16)	−0.00066 (15)
O1	0.0652 (13)	0.0641 (13)	0.0718 (14)	0.0178 (11)	0.0039 (11)	−0.0198 (11)
O2	0.0707 (14)	0.0654 (13)	0.0651 (14)	0.0163 (11)	0.0085 (11)	−0.0224 (11)
O3	0.0625 (13)	0.0630 (12)	0.0574 (12)	−0.0164 (10)	0.0107 (10)	0.0014 (10)
O4	0.0693 (13)	0.0660 (13)	0.0569 (12)	−0.0244 (11)	0.0195 (10)	−0.0107 (10)
S1	0.0502 (4)	0.0529 (4)	0.0540 (4)	0.0037 (3)	0.0124 (3)	−0.0015 (3)
S2	0.0501 (4)	0.0523 (4)	0.0540 (4)	0.0045 (3)	0.0146 (3)	0.0007 (3)
S3	0.0488 (4)	0.0529 (4)	0.0534 (4)	0.0041 (3)	0.0141 (3)	−0.0008 (3)
S4	0.0503 (4)	0.0528 (4)	0.0531 (4)	0.0035 (3)	0.0135 (3)	−0.0010 (3)

### Geometric parameters (Å, º)

**Table:**

C1—N1	1.335 (4)	C14—N2	1.314 (4)
C1—C2	1.348 (4)	C14—H14A	0.9599
C1—H1A	0.9601	C15—N2	1.430 (4)
C2—C3	1.340 (4)	C15—C16	1.502 (4)
C2—H2A	0.9600	C15—H15A	0.9601
C3—C4	1.331 (4)	C15—H15B	0.9599
C3—H3A	0.9599	C16—O3	1.163 (3)
C4—C5	1.372 (5)	C16—O4	1.338 (4)
C4—H4A	0.9600	C17—O4	1.471 (4)
C5—N1	1.338 (4)	C17—C18	1.478 (5)
C5—H5A	0.9601	C17—H17A	0.9600
C6—N1	1.450 (4)	C17—H17B	0.9598
C6—C7	1.506 (4)	C18—H18A	0.9600
C6—H6A	0.9600	C18—H18B	0.9600
C6—H6B	0.9599	C18—H18C	0.9600
C7—O1	1.200 (4)	C19—C21	1.349 (4)
C7—O2	1.271 (4)	C19—C20	1.417 (4)
C8—C9	1.482 (5)	C19—S1	1.723 (3)
C8—O2	1.572 (4)	C20—N3	1.148 (4)
C8—H8A	0.9600	C21—C22	1.422 (4)
C8—H8B	0.9600	C21—S2	1.737 (3)
C9—H9A	0.9600	C22—N4	1.132 (4)
C9—H9B	0.9600	C23—C25	1.358 (4)
C9—H9C	0.9600	C23—C24	1.435 (4)
C10—N2	1.325 (4)	C23—S3	1.719 (3)
C10—C11	1.375 (5)	C24—N5	1.138 (4)
C10—H10A	0.9600	C25—C26	1.415 (4)
C11—C12	1.369 (5)	C25—S4	1.730 (3)
C11—H11A	0.9600	C26—N6	1.136 (4)
C12—C13	1.310 (5)	Ni1—S4	2.1599 (9)
C12—H12A	0.9601	Ni1—S2	2.1636 (9)
C13—C14	1.329 (4)	Ni1—S1	2.1647 (9)
C13—H13A	0.9600	Ni1—S3	2.1714 (9)
			
N1—C1—C2	120.2 (3)	C16—C15—H15A	110.0
N1—C1—H1A	119.7	N2—C15—H15B	108.1
C2—C1—H1A	120.1	C16—C15—H15B	109.4
C3—C2—C1	120.4 (3)	H15A—C15—H15B	108.4
C3—C2—H2A	119.7	O3—C16—O4	125.5 (3)
C1—C2—H2A	119.9	O3—C16—C15	126.4 (3)
C4—C3—C2	120.0 (3)	O4—C16—C15	108.1 (2)
C4—C3—H3A	120.1	O4—C17—C18	106.1 (2)
C2—C3—H3A	119.8	O4—C17—H17A	109.7
C3—C4—C5	119.5 (3)	C18—C17—H17A	109.9
C3—C4—H4A	121.0	O4—C17—H17B	112.1
C5—C4—H4A	119.4	C18—C17—H17B	109.7
N1—C5—C4	120.1 (3)	H17A—C17—H17B	109.3
N1—C5—H5A	119.2	C17—C18—H18A	112.8
C4—C5—H5A	120.6	C17—C18—H18B	107.7
N1—C6—C7	114.1 (3)	H18A—C18—H18B	109.5
N1—C6—H6A	107.8	C17—C18—H18C	107.9
C7—C6—H6A	108.0	H18A—C18—H18C	109.5
N1—C6—H6B	109.2	H18B—C18—H18C	109.5
C7—C6—H6B	109.5	C21—C19—C20	120.3 (3)
H6A—C6—H6B	108.0	C21—C19—S1	119.6 (2)
O1—C7—O2	127.2 (3)	C20—C19—S1	120.1 (2)
O1—C7—C6	121.4 (3)	N3—C20—C19	178.8 (4)
O2—C7—C6	111.2 (3)	C19—C21—C22	120.5 (3)
C9—C8—O2	96.4 (3)	C19—C21—S2	121.3 (2)
C9—C8—H8A	104.2	C22—C21—S2	118.1 (2)
O2—C8—H8A	110.7	N4—C22—C21	178.1 (4)
C9—C8—H8B	116.9	C25—C23—C24	121.1 (3)
O2—C8—H8B	115.5	C25—C23—S3	121.4 (2)
H8A—C8—H8B	111.7	C24—C23—S3	117.4 (2)
C8—C9—H9A	108.3	N5—C24—C23	178.6 (4)
C8—C9—H9B	108.8	C23—C25—C26	122.1 (3)
H9A—C9—H9B	109.5	C23—C25—S4	119.8 (2)
C8—C9—H9C	111.3	C26—C25—S4	117.9 (2)
H9A—C9—H9C	109.5	N6—C26—C25	179.2 (4)
H9B—C9—H9C	109.5	C1—N1—C5	119.8 (3)
N2—C10—C11	119.5 (3)	C1—N1—C6	118.6 (3)
N2—C10—H10A	119.6	C5—N1—C6	121.5 (3)
C11—C10—H10A	120.9	C14—N2—C10	119.7 (3)
C12—C11—C10	118.4 (3)	C14—N2—C15	121.4 (3)
C12—C11—H11A	120.3	C10—N2—C15	119.0 (3)
C10—C11—H11A	121.3	S4—Ni1—S2	176.02 (3)
C13—C12—C11	120.3 (3)	S4—Ni1—S1	88.30 (3)
C13—C12—H12A	120.5	S2—Ni1—S1	91.58 (3)
C11—C12—H12A	119.2	S4—Ni1—S3	91.96 (3)
C12—C13—C14	119.5 (3)	S2—Ni1—S3	88.28 (3)
C12—C13—H13A	119.0	S1—Ni1—S3	178.30 (3)
C14—C13—H13A	121.5	C7—O2—C8	119.8 (2)
N2—C14—C13	122.5 (3)	C16—O4—C17	114.6 (2)
N2—C14—H14A	117.9	C19—S1—Ni1	104.30 (11)
C13—C14—H14A	119.6	C21—S2—Ni1	103.18 (11)
N2—C15—C16	111.5 (2)	C23—S3—Ni1	103.07 (10)
N2—C15—H15A	109.4	C25—S4—Ni1	103.71 (10)
			
N1—C1—C2—C3	0.5 (5)	C13—C14—N2—C10	−3.8 (5)
C1—C2—C3—C4	0.9 (6)	C13—C14—N2—C15	176.6 (3)
C2—C3—C4—C5	−2.1 (6)	C11—C10—N2—C14	3.5 (5)
C3—C4—C5—N1	2.1 (6)	C11—C10—N2—C15	−176.9 (3)
N1—C6—C7—O1	7.7 (5)	C16—C15—N2—C14	−79.4 (4)
N1—C6—C7—O2	−176.5 (3)	C16—C15—N2—C10	101.0 (4)
N2—C10—C11—C12	−0.7 (5)	O1—C7—O2—C8	15.0 (6)
C10—C11—C12—C13	−2.0 (5)	C6—C7—O2—C8	−160.4 (3)
C11—C12—C13—C14	1.8 (5)	C9—C8—O2—C7	−107.1 (3)
C12—C13—C14—N2	1.1 (5)	O3—C16—O4—C17	2.3 (5)
N2—C15—C16—O3	7.5 (5)	C15—C16—O4—C17	−177.4 (3)
N2—C15—C16—O4	−172.8 (3)	C18—C17—O4—C16	157.6 (3)
C20—C19—C21—C22	3.0 (5)	C21—C19—S1—Ni1	0.1 (3)
S1—C19—C21—C22	−175.5 (3)	C20—C19—S1—Ni1	−178.4 (2)
C20—C19—C21—S2	179.8 (2)	S4—Ni1—S1—C19	174.96 (12)
S1—C19—C21—S2	1.3 (4)	C19—C21—S2—Ni1	−2.0 (3)
C24—C23—C25—C26	8.0 (5)	S1—Ni1—S2—C21	1.54 (12)
S3—C23—C25—C26	−175.3 (3)	S3—Ni1—S2—C21	179.84 (12)
C24—C23—C25—S4	−177.9 (2)	C25—C23—S3—Ni1	−0.2 (3)
C2—C1—N1—C5	−0.5 (5)	C24—C23—S3—Ni1	176.6 (2)
C2—C1—N1—C6	−178.8 (3)	S4—Ni1—S3—C23	1.13 (11)
C4—C5—N1—C1	−0.8 (5)	C23—C25—S4—Ni1	2.0 (3)
C4—C5—N1—C6	177.4 (3)	C26—C25—S4—Ni1	176.3 (2)
C7—C6—N1—C1	−80.3 (4)	S1—Ni1—S4—C25	176.70 (12)
C7—C6—N1—C5	101.4 (4)	S3—Ni1—S4—C25	−1.61 (12)

### Hydrogen-bond geometry (Å, º)

**Table:**

*D*—H···*A*	*D*—H	H···*A*	*D*···*A*	*D*—H···*A*
C10—H10*A*···O1^i^	0.96	2.13	3.064 (4)	164
C13—H13*A*···N6^ii^	0.96	2.57	3.357 (4)	140
C15—H15*B*···S2	0.96	2.86	3.680 (3)	145

Symmetry codes: (i) −*x*, −*y*+1, −*z*+1; (ii) *x*+1, *y*, *z*.
